# Pyric tree spatial patterning interactions in historical and contemporary mixed conifer forests, California, USA

**DOI:** 10.1002/ece3.7084

**Published:** 2020-12-25

**Authors:** Justin P. Ziegler, Chad M. Hoffman, Brandon M. Collins, Eric E. Knapp, William (Ruddy) Mell

**Affiliations:** ^1^ Department of Forest & Rangeland Stewardship Colorado State University Fort Collins CO USA; ^2^ Center for Fire Research & Outreach University of California Berkeley CA USA; ^3^ Pacific Southwest Research Station US Forest Service Davis CA USA; ^4^ Pacific Southwest Research Station US Forest Service Redding CA USA; ^5^ Pacific Wildland Fire Sciences Laboratory US Forest Service Seattle WA USA

**Keywords:** complex adaptive system, fire frequent forest, fire‐caused mortality, forest restoration, physics‐based fire modeling, point pattern analysis, Wildland fire

## Abstract

Tree spatial patterns in dry coniferous forests of the western United States, and analogous ecosystems globally, were historically aggregated, comprising a mixture of single trees and groups of trees. Modern forests, in contrast, are generally more homogeneous and overstocked than their historical counterparts. As these modern forests lack regular fire, pattern formation and maintenance is generally attributed to fire. Accordingly, fires in modern forests may not yield historically analogous patterns. However, direct observations on how selective tree mortality among pre‐existing forest structure shapes tree spatial patterns is limited. In this study, we (a) simulated fires in historical and contemporary counterpart plots in a Sierra Nevadan mixed‐conifer forest, (b) estimated tree mortality, and (c) examined tree spatial patterns of live trees before and after fire, and of fire‐killed trees. Tree mortality in the historical period was clustered and density‐dependent, because trees were aggregated and segregated by tree size before fire. Thus, fires maintained an aggregated distribution of tree groups. Tree mortality in the contemporary period was widespread, except for dispersed large trees, because most trees were a part of large, interconnected tree groups. Thus, postfire tree patterns were more uniform and devoid of moderately sized tree groups. Postfire tree patterns in the historical period, unlike the contemporary period, were within the historical range of variability identified for the western United States. This divergence suggests that decades of forest dynamics without significant disturbances have altered the historical means of pyric pattern formation. Our results suggest that ecological silvicultural treatments, such as forest restoration thinnings, which emulate qualities of historical forests may facilitate the reintroduction of fire as a means to reinforce forest structural heterogeneity.

## Graphical abstract

Fire effects on the spatial arrangement of trees have shifted dramatically in a mixed conifer forest over the course of 80 years. In 1929, fires maintained the clustered, patchy distribution of trees, lowering the average size of tree groups. In 2008, severe fires spread throughout the homogeneous forest canopy, yielding few and scattered trees.

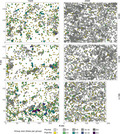

## 1 INTRODUCTION

Drier mixed conifer forests of western North America have long been shaped by frequent fire. These fires mediated heterogeneous, uneven‐aged forest structures at fine scales through partial and periodic tree mortality, stimulating fire‐adapted understory plants, and creating temporally and spatially variable conditions for tree regeneration (Knapp et al., 2013; Larson & Churchill, 2012; Show & Kotok, 1924). Consequently, forest structure was patterned into complex mosaics composed of scattered individual trees, groups of trees, and canopy openings occupied by understory plants or regenerating trees (Larson & Churchill, 2012). Formation of tree groups was spurred by conditions such as small gaps with higher light availability, patchy distributions of mineral soil exposed by fire (Larson & Churchill, 2012), microclimate amelioration by neighbors and nurse trees (Fajardo et al., 2006), and zoochoric seed caching (Vander Wall & Joyner, 1998). Canopy openings were likely a product of either localized agents of tree mortality, unfavorable microsite conditions for tree regeneration such as shallow soils (North et al., 2004), or resource competition with nontree species (Abella et al., 2013).

Historical fire behavior and effects likely varied at fine scales in response to heterogeneously patterned forest structure and composition. Surface fuels accumulate in groups with more tree basal area (Banwell & Varner, 2014), and local crowding within tree groups increases the probability of intertree fire spread (Contreras et al., 2012). This may have led to a clustered pattern of tree mortality, especially in areas with dense tree groups (Hood et al., 2018; Larson & Churchill, 2012; Lutz et al., 2018). Alternatively, fire severity may increase in openings due to drier and windier microclimates (Bigelow & North, 2012) and the contribution of greater understory cover to surface fuel loads (Stephens et al. 2021, Matonis & Binkley, 2018). Thus, locations of higher severity patches may have been dispersed and in areas with lower tree stocking. Inferences regarding fine‐scale pyric regulation of forest structure are often based on comparisons of tree spatial patterns in contemporary, fire‐suppressed forests against historical forests or contemporary forests with intact fire regimes (e.g., Fry et al., 2014; Schneider et al., 2016). However, the lack of direct fire observation in these and other studies makes it difficult to understand the pattern–process interactions driving pyric regulation. Even when measurements are made before and after fires on individual sites, it is challenging to separate fire effects from other co‐occurring processes such as density‐dependent competition (Yu et al., 2009; but see Furniss et al., 2020).

Recently, physics‐based fire modeling has been suggested as an ideal approach to test conceptual models of fire‐mediated forest dynamics (Lutz et al., 2018) because simulations allow for a high degree of experimental design and control (Hoffman et al., 2018; Larson & Churchill, 2012; Lutz et al., 2018; Parsons et al., 2017). This line of inquiry has explored the feedback between heterogenous fuel arrangements and consequent fire behavior across stands (Hoffman et al., 2012; Linn et al., 2013; Parsons et al., 2017) and distance‐dependency of tree‐to‐tree crown fire spread (Contreras et al., 2012). However, relatively few studies have used physics‐based fire modeling to explore how fire interacts with forest structure patterns within stands (e.g., Ritter et al., 2020). Furthermore, an explicit comparison of how pattern–process interactions may differ between historical and contemporary forests is lacking.

**Figure 1:**
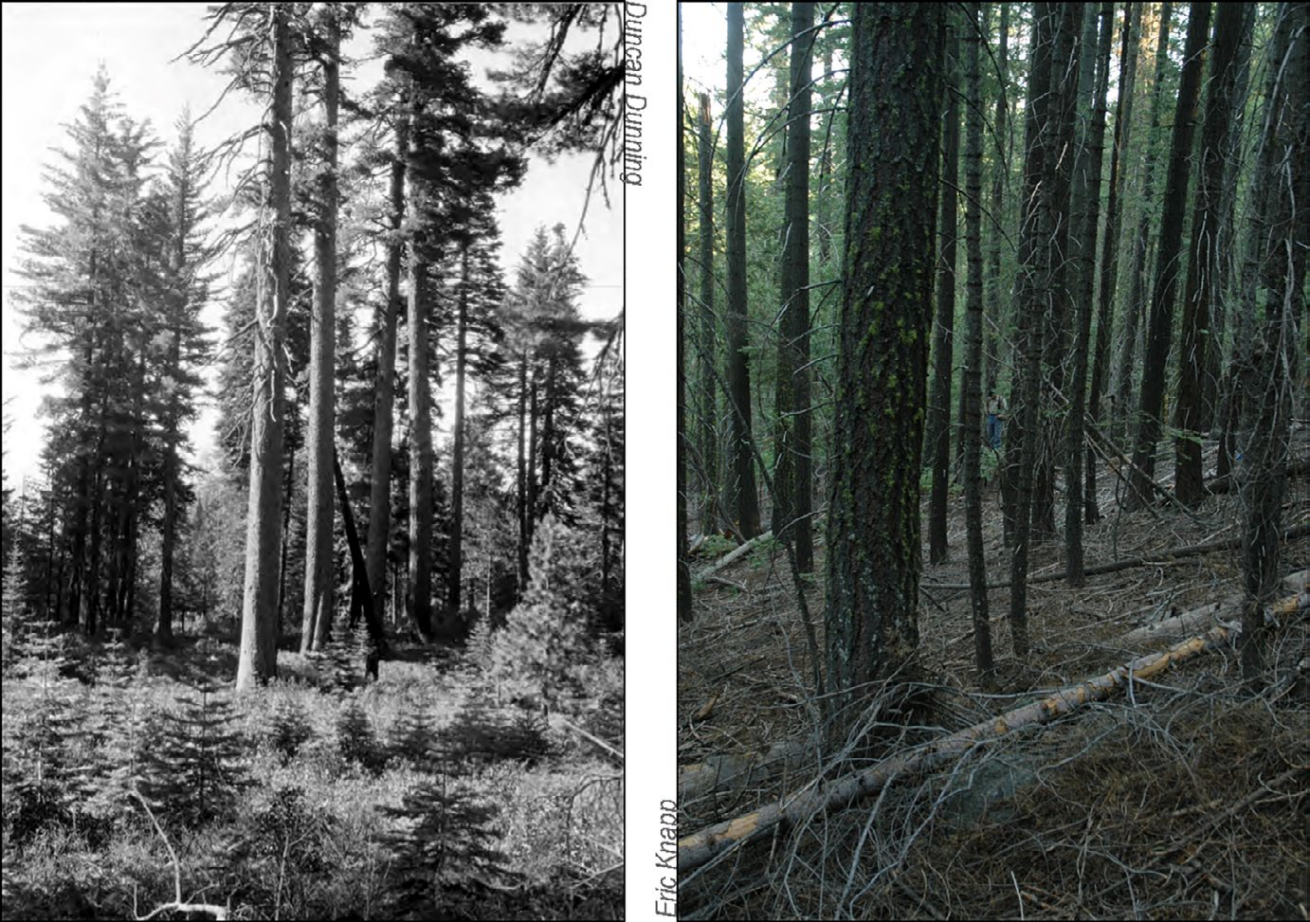
Photographs of the mixed conifer forest in one of this study's plots contrasting low canopy cover and a heterogeneous overstory and understory in 1929 (left) versus high canopy cover and a homogeneous overstory and understory in 2007 (right photograph)

An increased understanding of fine‐scale fire‐structure interactions can guide fuel hazard reduction treatments. This knowledge is particularly pertinent for forest restoration treatments that emulate historical forests' qualities, expressly creating a heterogeneous structure composed of single trees and tree groups (Knapp et al., 2017; Tuten et al., 2015; Ziegler et al., 2019). Over a century of fire exclusion, as well as unregulated grazing and logging, have increased tree densities, reduced the number and size of openings, favored shade‐tolerant species, and decreased heterogeneity of the over and understory (Figure 1; Iniguez et al., 2019). Changes in forest structure and composition have resulted in greater and more uniform canopy and surface fuel loads (Fry et al., 2014; Lydersen et al., 2013; Matonis & Binkley, 2018) and increased fire behavior (Hessburg et al., 2005). If the spatial patterns of trees influence the distribution of fire effects, the loss of fine‐scale structural variability may be dampening the pattern–process relationship once present in historical forests (Hessburg et al., 2005; Parsons et al., 2017). Although forest restoration treatments seek to restore such relationships (Addington et al., 2018; Larson & Churchill, 2012; Tuten et al., 2015; Ziegler et al., 2017), treatments leaving evenly spaced trees continue to be implemented (Puettmann et al., 2015; Stephens et al., 2021; Underhill et al., 2014), based on aspatial fire hazard reduction principles (Larson & Churchill, 2012). Even within some variable retention harvesting methods, specifications often implement spacing‐based targets within tree groups (Tuten et al., 2015). A greater understanding of how fine‐grained forest overstory and understory structure interacts with fire to mediate tree patterning can help aid the design and evaluation of restoration‐based silvicultural approaches (Knapp et al., 2017; Lutz et al., 2018). Additionally, this information may provide insight into the link between pattern and process at fine spatial scales and its stability over a century of forest change.

In this study, we examined the spatial dynamics of fire‐caused mortality across a time series in a mixed conifer forest, which historically experienced frequent fire. We leveraged data from three large (~4 ha) forest plots that were stem‐mapped immediately before harvesting in 1929, approximating forests before EuroAmerican settlement characteristics, and again in 2008, representative of contemporary long‐unburned forests with a history of logging. We used a physics‐based fire model to simulate fire spread in each of the two time periods and then estimated fire‐caused mortality based on species and tree size. We hypothesized that mortality would be clustered and density‐dependent in the historical period due to pre‐existing spatial variability typical of historical forests; we further hypothesized the residual forest structure would retain a mosaic of tree groups of diverse sizes. In contrast, we expected that mortality patterns would be more random and density‐independent because the 2008 counterpart plots were comparatively homogeneous with continuous canopy as opposed to discrete tree groups (Lydersen et al., 2013). Such random mortality patterns might then leave behind a less variable distribution of tree group sizes. We recognize that factors in addition to tree mortality—namely spatially variable regeneration dynamics driven by spatially variable abiotic conditions—also contributed to the historical pattern.

## 2 MATERIAL AND METHODS

### 2.1 Study area

We used three large plots of the permanent “Methods of Cutting” study established in 1929 in the Stanislaus‐Tuolumne Experimental Forest of the central Sierra Nevada. The study sites have a Mediterranean climate, with warm, dry summers and cool, moist winters (Knapp et al., 2013). The sites are on a northwest aspect, at an elevation from 1,805 m to 1,840 m above sea level, and on deep, well‐drained gravelly loam soil (Knapp et al., 2013; Lydersen et al., 2013). These plots, named MC9 (4.3 ha area), MC10 (3.8 ha), and MC11 (4.3 ha), were originally designed to investigate regeneration and growth rates following silvicultural prescriptions in a mixed conifer forest dominated by *Abies concolor* Lindl. ex Hildebr., *Pinus lambertiana* Douglas, *Calocedrus decurrens* Florin, *P. ponderosa* ex. Lawson, and *P. jeffreyi* Balf., in order of abundance. Locations, species, heights, and diameter at breast height (dbh) of trees ≥ 9.1 cm dbh were recorded in 1929 prior to partial harvesting. The understory fuels were mapped into broad cover type patches (rock, tree regeneration, understory shrubs by dominant species, and the remainder assumed to be forest litter). Stem mapping of trees ≥ 10 cm dbh in MC9 and MC10 occurred again in 2008. In 2007, we remapped only 3.4 ha in MC11 because a road had built through it. For brevity, we refer to the 2007 and 2008 measurements as the 2008 measurement period. Additional information on the history of these plots and prior research is provided in Hasel et al. (1934), Knapp et al. (2013), and Lydersen et al. (2013).

Prior work from Knapp et al. (2013) and Lydersen et al. (2013) have examined the forest structures in 1929 and 2008, placing these sites in the context of historical reference conditions, and contemporary conditions among similar forests. Results from these studies suggest the two sampling periods were representative proxies for pre‐EuroAmerican and once‐logged contemporary forests in mixed conifer forests of the Sierra Nevada. It is worth noting the historical median and maximum fire return intervals were six years and forty years, respectively; the last fire occurred in 1889, forty years before sampling (Knapp et al., 2013). Forest structure sampled in 1929 therefore represented the upper end of the historical range of variability regarding fire frequency. Consequently, our exclusion of smaller trees from sampling in 1929 may have overlooked tree regeneration establishing during the longer fire‐free interval; meanwhile, few trees were likely omitted in 2008 because tree regeneration was sparse at that time (Knapp et al., 2013; Figure 1).

### 2.2 Fire behavior modeling

We simulated fire behavior with the Wildland‐urban interface Fire Dynamics Simulator. Using a computational fluid dynamics approach, numerical solutions are solved in a domain composed of discretized voxels over a series of time steps (Mell et al., 2007, 2009). This approach allows for the representation of vegetation and fire behavior over three‐dimensional space and time. We simulated fire spread in six instances (each of the three plots over two time periods), using the respective stem maps and accompanying understory cover type. Each of the understory cover types was crosswalked to a standard surface fuel model (Scott & Burgan, 2005). The four cover types mapped initially in 1929, included conifer litter, tree regeneration, *Chamaebatia foliolosa* (Benth.) shrubs, and shrubs of other species, represented by standard surface fuel models timber‐litter 3 (TL3), timber‐litter 1 (TL1), grass‐shrub 2 (GS2), and shrub 2 (SH2), respectively. Because understory vegetation had almost entirely disappeared by 2008 as gaps in the forest filled with trees, we simulated those surface fuels as a homogenous layer of TL3.

We simulated relatively high fire weather conditions because more severe burning conditions were expected to elicit tree mortality. Furthermore, high to extreme fire weather conditions are associated with a majority of burned area in western U.S. wildland fires (Finney et al., 2011). Wind speeds entering the domain were set at 5.07 m/s at 6.1 m above ground level. Surface and crown fuel moistures were simulated at 5% and 100%, respectively. These values represent the 99.9th and 14.5th percentile for the wind speed and 1‐hr dead downed woody fuel moisture, respectively, compared to data from 2011–2019 at the nearby Pinecrest 2 remote automated weather station (National Weather Service ID #043615). Appendix S1 gives technical detail on the design and further parameterization of the simulations.

We calculated gross and per‐tree crown consumption (percent dry mass lost) from simulation results. We used these results to estimate mortality following Parsons et al. (2018); we applied tree mortality likelihood equations from Hood et al. (2007) using dbh, tree species, and, in lieu of percent crown volume scorched, crown consumption. Trees with a mortality likelihood *≥* 50% were designated fire killed.

### 2.3 Point pattern analyses

We used a framework of point pattern analyses to examine stand‐scale spatial patterns of trees before each fire, and the spatial dynamics of projected mortality following each fire. All statistical inferences were made using an *α* = 0.05. Point pattern analyses were conducted in Programita ver. 2018 (Wiegand & Moloney, 2013). We used tidyverse ver. 1.2.1 (Wickham et al., 2019) for data wrangling, and cowplot ver. 1.0.0 (Claus & Wilke, 2019) and ggthemes ver. 4.2.0 (Arnold, 2019) for data visualization, in R ver. 3.6.1 (R Core Team, 2019).

### 2.4 Mark correlation functions

We used mark correlation functions to describe the spatial structure of tree sizes, aiding interpretation of fire effects. Mark correlation functions yield statistics of an appropriate test function averaged over all pairs of trees at distance *r* apart. We first used the *r*‐mark correlation function (Illian et al., 2008), termed *k*_dbh_(*r*) here, whose test statistic was the average tree dbh located *r* away from another tree. By comparing the empirical statistics to a null model of random labeling where dbh is randomly shuffled among tree locations, we could assess whether trees distanced *r* away from another tree were smaller or larger than the mean aspatial tree dbh. In addition, we used a mark variogram, *γ*_dbh_(*r*), to assess whether tree sizes were spatially correlated. The test function $\frac{1}{2}{\left({\mathrm{dbh}}_{i}‐{\mathrm{dbh}}_{j}\right)}^{2}$ described the semivariance in tree dbh between two trees, *i* and *j*, located *r* distance apart. We compared the empirical statistics to a null model of random labeling to determine whether dbh between trees located *r* distance apart were more or less variable than expected by chance.

### 2.5 Pair correlation functions

The first pair of univariate pair correlation functions described the patterns of living trees, both before fire, *g*_all_(*r*), and after fire, *g*_alive_(*r*). Univariate pair correlation functions measure the average number of points, that is, trees, at distance *r* from a point, normalized by dividing by the expected number of points. The empirical pair correlation functions are compared to a set of functions realized from a null model. In these analyses, we used an inhomogeneous Poisson point process model. This null model randomly distributed points using an intensity field parameterized by an Epanechnikov smoothing kernel at a bandwidth of 20 m and a resolution of 1 m. We chose an inhomogeneous over a homogeneous Poisson process to account for intensity gradients in the observed data. Any values above or below expectation reflected that trees were spatially distributed as aggregated or uniform patterns, respectively.

The next pair of pair correlation functions described the patterns of estimated fire‐killed trees. Bivariate pair correlation functions count the average number of type 2 points at distance *r* from a type 1 point, normalized by the density (points per area) of type 2 points (Wiegand & Moloney, 2013). Here, the types were the labels of alive or dead. First, we calculated the difference *g*_dead,dead_(*r*)‐ *g*_alive,dead_(*r*), concisely referred to here as *g*_cluster_(*r*); *g*_dead,dead_(*r*) measured the relative density of fire‐killed trees near fire‐killed trees and *g*_alive,dead_(*r*), the relative density of fire‐killed trees near surviving trees. Thus, *g*_cluster_(*r*) estimated whether fire‐killed trees were more, less, or equally common around surviving trees than around other fire‐killed trees. The null expectation was 0, whereas higher values indicated clustering of mortality, and lower values indicated the dispersion of mortality. Second, we measured density dependence of mortality with the difference *g*_dead,dead+alive_(*r*)*−g*_alive,dead+alive_(*r*). This compared the relative density of all trees near a dead tree minus the relative density of all trees near a surviving tree. The null expectation was 0; higher and lower values indicated a bias toward mortality in areas of higher or lower density, respectively. For the null models of *g*_cluster_(*r*) and *g*_dens.dep_(*r*), we randomly labeled points’ types, alive or dead, rather than moving points because the locations of survival or mortality are conditioned on the spatial pattern of trees before a fire (Goreaud & Pelissier, 2003).

Monte Carlo methods were used to create realizations for assessing the departure of empirical statistics from a null model. We generated 399 simulations of the respective null models for each point pattern analysis over a range of *r* from 0 to 15 m. The range of *r* should reflect the scale that spatial correlations are expected to manifest a priori, often 0—15 m among trees (Wiegand & Moloney, 2013). A simulation envelope can be constructed from the 2.5th to 97.5th percentile statistic for each distance *r* followed by a comparison of the empirical statistic against the envelope (Wiegand & Moloney, 2013). However, standardization is recommended to make formal inference without inflated Type I Error (Wiegand & Moloney, 2013). Therefore, we first studentized the empirical and null correlation functions to *z* scores, constructed maximal global envelopes from the 2.5 to 97.5 percentiles, and then back transformed those functions’ statistics to their original scale (Myllymäki et al., 2015). Under this approach, a deviation at any distance r from the envelope constituted a statistically significant deviation for the set of *r* from 0 to 15 m (Wiegand & Moloney, 2013). Last, we then averaged mark correlation functions and pair correlation functions across distance *r* from 0 to 15 m to produce a single statistic to compare the strength of departure from null models across time periods. Our implementation of point pattern analyses followed best practices laid out in Velázquez et al. (2016); we accounted for edge effects.

### 2.6 Modeling fire effects on within‐stand structure

We tested whether fires produced different outcomes in the distributions of tree group size (number of trees per group) in 1929 versus 2008. Tree groups were identified following the approach whereby trees within a group are within a limiting distance, here 6 m, from another member tree (e.g., Lydersen et al., 2013). First, we compared the median group sizes with a Wilcoxon signed rank test, and, second, the variation of group sizes with a modified signed‐likelihood ratio test for equality of coefficients of variation (SLRT). We modified *p* values with a Bonferroni correction to account for multiple comparisons across the three plots. Third, we used Sankey diagrams to visualize how trees’ group sizes changed due to fires. For this, we binned groups into size classes: single trees, 2–4, 5–9, 10–19, and 20+ trees per group.

## 3 RESULTS

### 3.1 General forest structure and fire behavior

**Figure 2:**
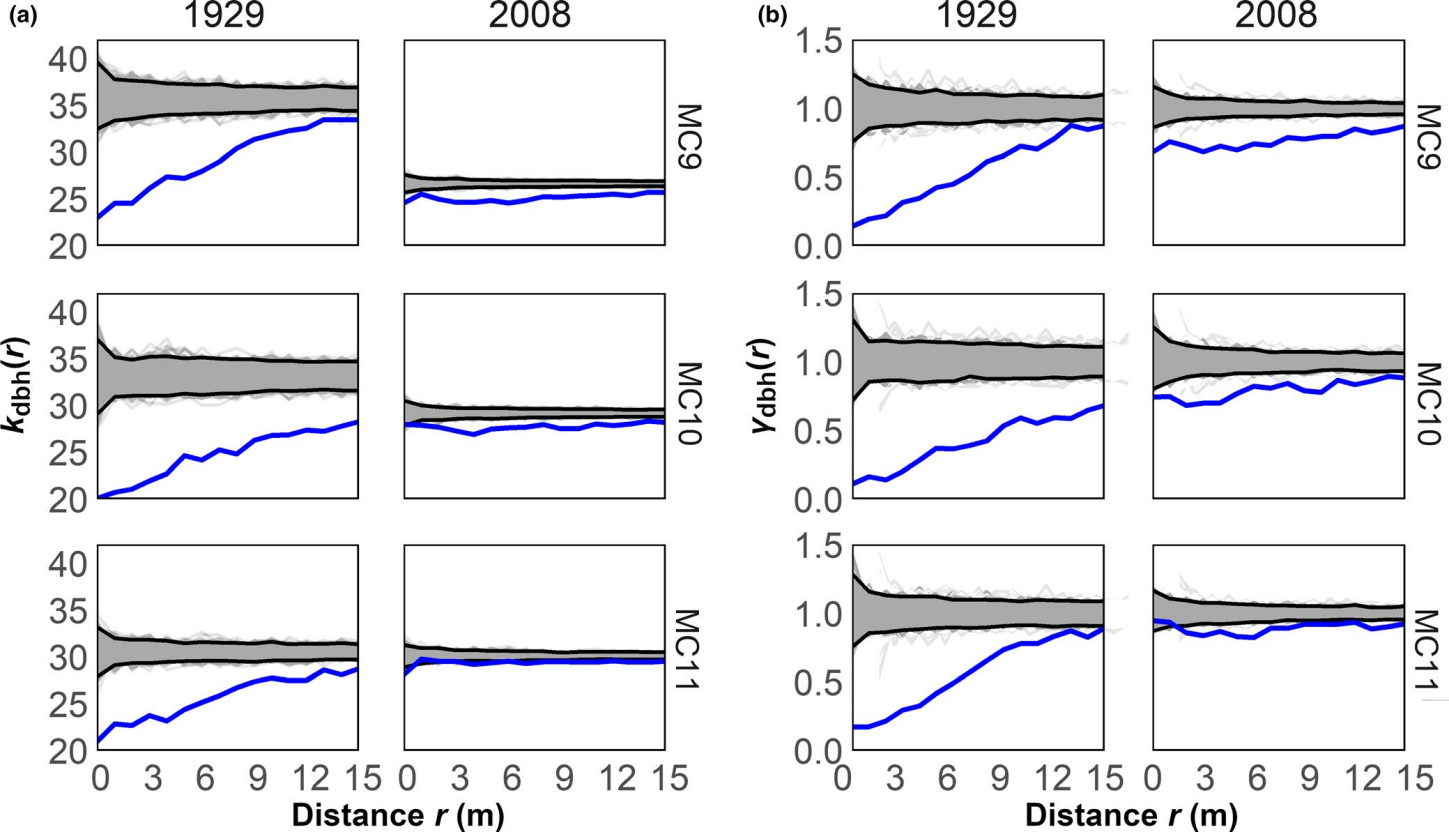
Mark correlation functions describing the spatial structure of tree diameter at breast height (dbh) in 1929 and 2008 for each plot (MC9, MC10, MC11) where (a) shows the r‐mark correlation function, *k*_dbh_ *(r)*, which is the average dbh at distance *r* from another tree and (b) shows the mark variogram, *γ*_dbh_ *(r)* which is the correlation of dbh between trees at distance *r* apart. Blue lines are the empirical functions, while gray lines are simulated functions of null models generated via random labeling and black lines are the 95th percentile confidence envelopes

Forest structure differed substantially between 1929 and 2008 in all three plots (Table 1). Despite trees being on average considerably smaller in 2008 than 1929, basal areas were higher in 2008 because tree density was greater. Furthermore, the canopy base height in 1929 was twice as tall on average as in 2008. Tree sizes were less randomly distributed in 1929 than in 2008. In 1929, the dbh of trees within 15 m of each other was smaller (26.6 cm averaged across plots) than the average dbh of all trees (33.1 cm averaged across plots; Figure 2a). Further, the semivariance of those trees’ dbh within 15 m of another averaged 0.51 across plots (Figure 2b). In contrast, the dbh of trees within 10 m of each other in 2008 averaged 27.4 cm, while the average dbh of all trees was 28.7 cm (Figure 2a). In addition, the semivariance of those trees’ dbh within 15 m of another averaged 0.82 across plots (Figure 2b). These results show that trees closer to one another tended to be similar in size and relatively smaller. This pattern was much more pronounced in 1929 than in 2008.

**Table 1:** Summary of stand structure, within and across species, by plot and measured year, as well as canopy consumption and predicted mortality

Year/Plot	Prefire forest structure				Rate of spread (m/s)	Canopy consumption (%)	Mortality (% of prefire stocking)	
	Trees per hectare	Basal area (m^2^/ha)	QMD (cm)	Canopy base height^a^ (m)			Trees per hectare	Basal area (m^2^/ha)
1929								
MC9	307	54.8	47.8	1.4	0.64	27	53	17
MC10	300	52.0	47.2	1.4	0.58	27	62	16
MC11	434	60.1	42.2	1.4	0.49	26	57	16
2008								
MC9	846	68.0	32.1	0.7	0.65	90	97	84
MC10	723	72.2	35.8	0.7	0.66	90	97	84
MC11	680	66.1	35.4	0.8	0.57	78	88	62

^a^ Canopy base height expressed as the tenth percentile crown base height.

Fire behavior and effects predictions were numerically higher in 2008 than in 1929. Averaged across plots, rates of spread were 0.57 m/s and 0.63 m/s in 1929 and 2008, respectively. Canopy consumption tripled from 1929 to 2008, averaging 26% and 78% in 1929 and 2008. Last, mortality estimates rose from 57% of trees in 1929 (16% of basal area) to over 94% of trees (77% of basal area) in 2008.

### 3.2 Fire effects on tree spatial patterns

**Figure 3:**
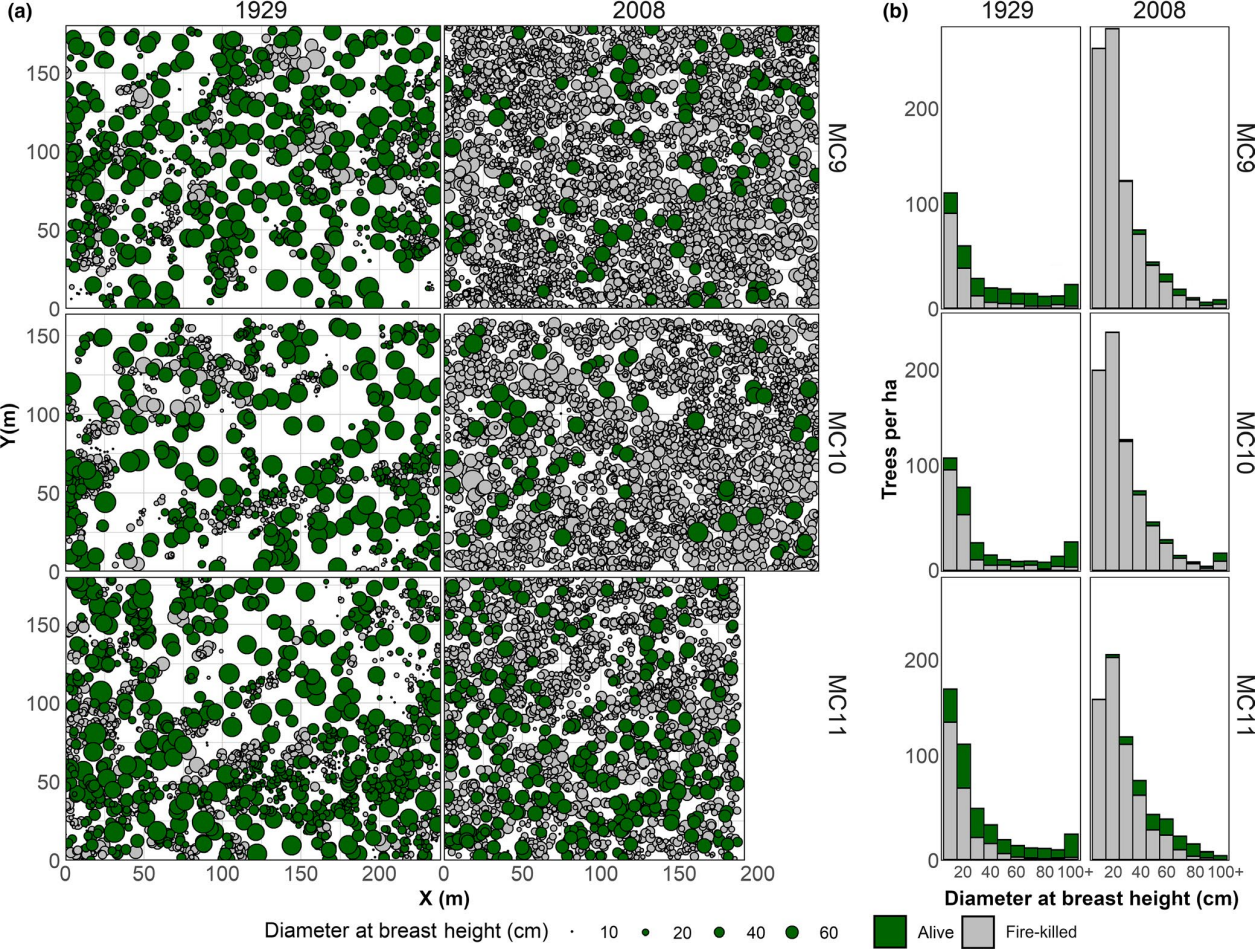
(a) Locations of killed and surviving trees, by plot and year, and (b) histograms of trees by size

Spatial and aspatial distributions of surviving and fire‐killed trees were markedly different across time periods. Whereas tree mortality and survival were arranged in a patchy mosaic in 1929, only scattered trees were estimated to survive in 2008 (Figure 3a). In both time periods, larger trees were more likely to survive (Figure 3b); across plots, in 1929, the average surviving tree ranged from 46.3 to 56.7 in 1929 and 59.8 cm to 81.2 cm dbh in 2008.

**Figure 4:**
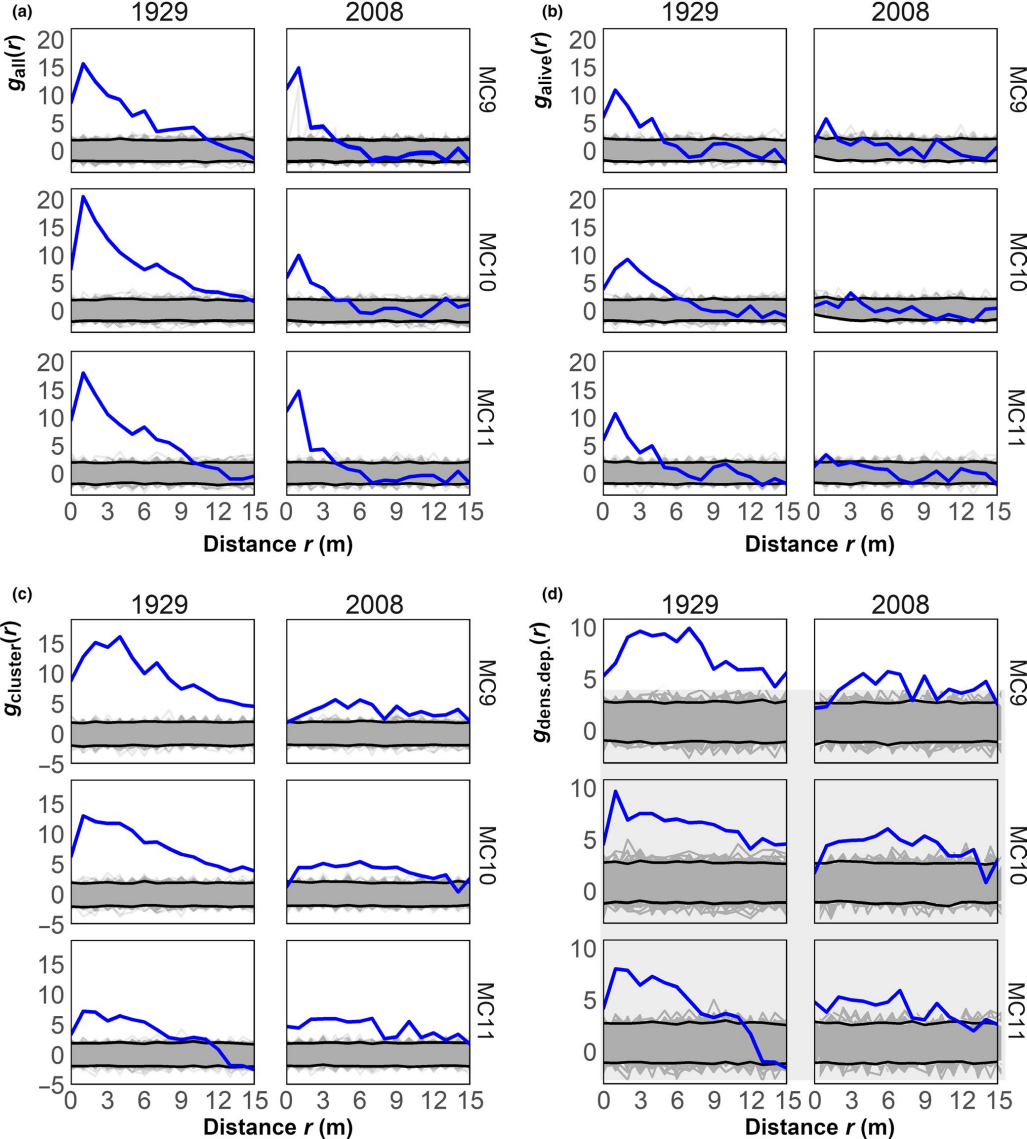
Standardized effect size of four points pattern statistics within plots in 1929 and 2008. The functions describe the spatial pattern of all trees before a simulated fire (*g*_all_(*r*); (a), the pattern of residual trees (*g*_alive_(*r*); (b), the clustering of killed trees (*g*_cluster_(*r*); (c), and the density dependence of killed trees (*g*_dens.dep_.(*r*); (d). Blue lines are the empirical functions, while gray lines are simulated functions of null models generated via random labeling, and black lines are the 95th percentile confidence envelopes

Simulated fires markedly altered the spatial patterns of trees in 1929. Trees were initially aggregated; as a measure of aggregation magnitude, *z* scores of *g*_all_ *(r)*, averaged across *r*, ranged from 5.4 to 7.6 across sites (Figure 4a). Aggregation was present postfire but diminished; *r*‐averaged *z* scores of *g*_alive_ *(r)* ranged from 1.8 to 2.4 (Figure 4b). Mortality was not randomly distributed (Figure 4c), with clustering of fire‐killed trees (*g*_cluster_(*r*); *r*‐averaged *z*: 3.1—9.6). Further, dead trees had more neighbors than surviving trees (*g*_dens.dep_.(*r*); Figure 4d), indicating mortality was density‐dependent (*r*‐averaged *z*: 3.1—6.4).

Trees were less aggregated in 2008 (*g*_all_ *(r)* *r*‐averaged *z*: 1.8—2.2*;* Figure 4e). Fires also dampened residual aggregation, measured by *g*_alive_ *(r)* (Figure 4f; *r*‐averaged *z*: 0.2—0.8). The spatial distributions of the fire‐killed trees were less clustered (Figure 4g; *r*‐averaged *z*: 3.7—4.3), as measured by *g*_cluster_(*r*). These killed trees were also in locations of higher tree density (*g*_dens.dep_.(*r*); *r*‐averaged *z*: 3.1—3.3). Compared to 1929, the magnitude of all measures—tree aggregation before and after a fire, and clustering and density dependence of fire‐killed trees—were all lower 2008.

### 3.3 Fire effects on tree groups

**Figure 5:**
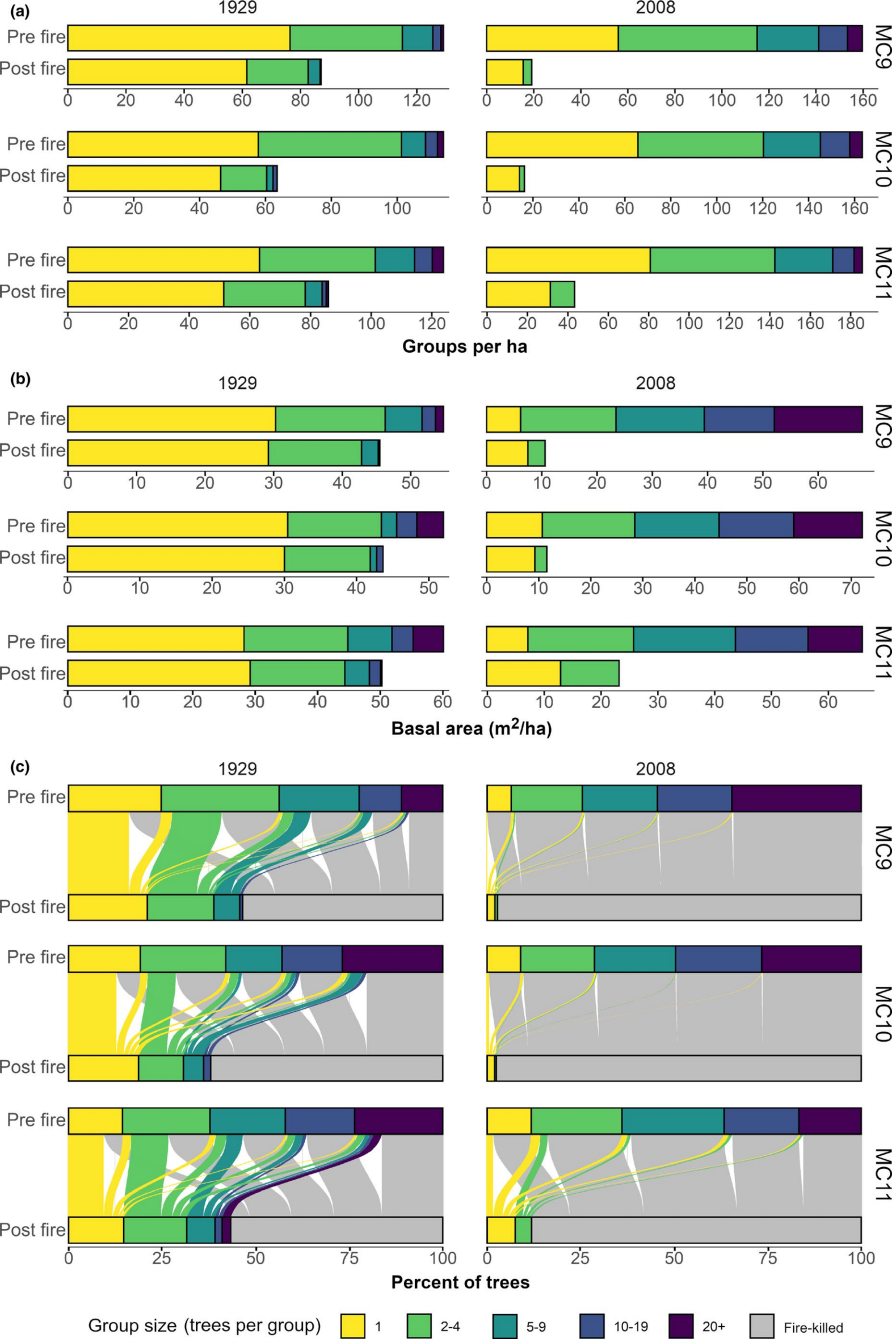
Change in tree groups per hectare (a), basal area by tree group (b), and flow of trees between tree group sizes (c), following simulated fire

Before fire, tree groups were more numerous and larger in 2008 than in 1929 (Figure 5). In 1929, there were between 114 and 129 groups per hectare. Groups with multiple trees amounted to 41% to 49% of all tree groups, constituting 75% to 85% of all trees and 41% to 53% of the stand basal area. In contrast, there were between 160 and 186 groups per hectare in 2008. Between 56% to 65% of these groups were multitree groups, with 88% to 93% of all trees and 85% to 91% of the stand basal area. The mean group size (inclusive of single trees) before fire was significantly smaller in 1929 (2.4–3.5 trees per group) than in 2008 (3.7–5.3 trees per group; Wilcox tests *p* values ≤ 0.03). The coefficient of variation (CV) of prefire tree group size in 1929 versus 2008 differed in MC9 (CV of 1.57 and 2.08, respectively; SLRT *p* = .03), but not in MC10 (CV of 2.26 and 1.72, respectively; SLRT *p* = .14), nor in MC11 (CV of 1.54 and 1.37, respectively; SLRT *p* = .72).

After fire, tree groups were fewer in number, smaller in size, and less variably sized in 2008 than 1929 (Figure 5). Plots in 1929 had 64 to 87 groups per hectare. 27% to 40% of groups, across plots, had multiple trees, accounting for 57% to 71% of trees and 31% to 43% of the basal area. After fire in 2008, there were 16 to 44 groups per hectare, with 13% to 28% of groups as multitree groups. These multitree groups made up 26% to 36% of trees and 20% to 44% of basal area. Residual tree groups averaged 1.5–1.9 and 1.1–1.3 trees per group in 1929 and 2008, respectively. The difference in group sizes between periods was supported by Wilcoxon tests in MC10 (*p* = .01) and MC11 (*p* < .01) but was not significantly different in MC9 (*p* = .07). Further, residual tree groups were less variable in size in 2008 (CV from 0.32 to 0.46) than 1929(CV from 0.79 to 1.16; SLRT, *p* values < 0.01).

**Figure 6:**
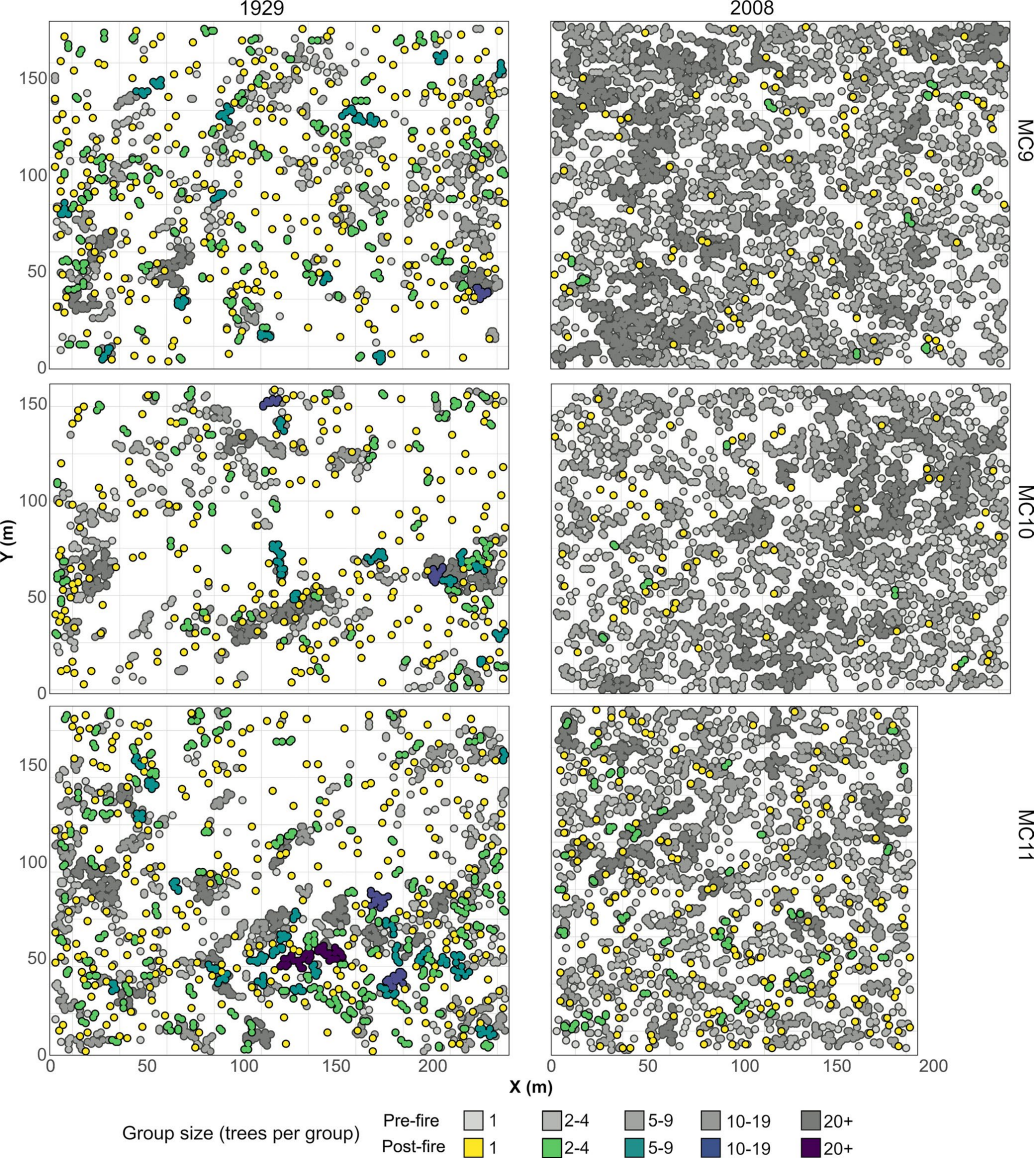
Mapped change in distribution of trees, by tree group size class

The effect of fires on tree group size distributions differed greatly between time periods. In 1929, trees within larger groups were more likely to be killed (Figure 5c). For example, approximately two thirds of all single trees survived, providing the bulk of single trees postfire, but less than half of trees in groups of 2 to 4 trees persisted. Fires, therefore, had the effect of splitting larger tree groups into smaller residual groups (Figure 6). In 2008, however, a large majority of trees were killed regardless of their respective tree group size (Figure 5c), and residual single trees and groups of 2–4 trees were derived from a mixture of all pre‐existing group sizes (Figures 5c,6).

## 4 DISCUSSION

Fine‐scale heterogeneity in forest structure is increasingly recognized as a salient characteristic of forests that historically experienced frequent fire (Clyatt et al., 2016; Larson & Churchill, 2012; Puettmann et al., 2015). This heterogeneity is thought to have been a self‐reinforcing pattern–process relationship with low to moderate severity fire (Bonnicksen & Stone, 1980; Larson & Churchill, 2012). Our modeling results based on the historical data support this interpretation and point to a strong local fuel control on fire effects. Simulated fires in 1929s forest condition maintained qualitative and quantitative characteristics typical of forests adapted to frequent fire. Specifically, trees predicted to survive these fires were arranged in an aggregated pattern (Figure 3b) consisting of both single trees and groups of up to 20 trees (Figure 5). This mirrors the patterns of trees reconstructed in many frequent‐fire forests of western North America before EuroAmerican settlement (Larson & Churchill, 2012). Clyatt et al. (2016) found that most (~73% to 99%) trees in this region were historically single trees or in small groups of 2 to 9 trees. We found fewer single trees or trees in small groups (64% on average) in the historical period before fire, whereas 94% of trees were single or in small groups after simulated fires. Though this shift was large—greatly reducing larger (10+ trees) groups and increased the relative abundance of single trees—the postfire forest structure fell within the historical range of variability reported by Clyatt et al. (2016).

Simulated fires in the contemporary forest condition produced very different patterns of surviving trees than those based on the historical forest condition. As has been identified in many other studies in frequent‐fire forests (e.g., Iniguez et al., 2019; Sánchez Meador et al., 2009), tree establishment and growth over decades without fire at our study site contributed to many more trees that were arranged in a more homogeneous condition (Lydersen et al., 2013). This coupling of higher tree density and greater homogeneity resulted in a relatively continuous tree canopy layer, which was quite different from the broken, clumpy tree canopy layer in the 1929 condition (Figure 3). After fire, no tree groups had more than 4 trees, and 64% to 82% of all trees were single trees. The shift from trees occurring mostly in large tree groups before fire to single trees after fire has been observed elsewhere in contemporary Sierra Nevada forests in patches of high severity (Kane et al., 2019). This pattern occurred because the surviving larger, more fire‐resistant, trees were dispersed rather than clustered, a common characteristic among the largest trees in many frequent‐fire forests (Boyden et al., 2005; Larson & Churchill, 2012). Consequently, our results suggest high‐severity fires in overstocked, contemporary forests are more likely to yield random patterns of sparse residual trees rather than rectify the trend toward homogenization over fire‐free decades.

Spatial patterns of predicted mortality from fire can be largely attributed to local arrangements of differently sized trees. In 1929, fire‐killed trees were generally smaller, highly clustered, and density‐dependent. This pattern is facilitated by the spatial segregation of trees by size class, leading to clustered mortality among smaller trees, which tend to be near each other (Figure 2). This pattern of clustered density‐dependent fire‐caused tree mortality has been observed in similar Sierran mixed conifer forests (Kane et al., 2019) and dry *Pinus sylvestris* (L. var. mongolica Litv.) forests in China (Yu et al., 2009). In contrast, fire‐killed trees in 2008 were widespread, not clustered, and less density‐dependent than in 1929. These differences are due to a combination of intermixed tree sizes, which is related to the dispersion of small trees, as well as higher tree stocking, larger tree groups, and fewer canopy interspaces (Figure 2, Figure 5). First, small tree dispersion provides numerous points for surface to crown fire transition to occur. Second, the increased stocking and presence of large tree groups reduces local convective cooling, facilitating both crown fire transition and spread (Ritter et al., 2020). These results suggest that the fire‐mediated patterns of tree mortality have been significantly altered since historical times and that these altered patterns are produced by more severe fires resulting from greater tree densities and altered tree arrangements.

Simulated fires in both periods involved the same fire weather scenario, which by most standards would be considered high fire danger (Bradshaw et al., 1984). Interestingly, despite this fire danger level, the forests in the historical period maintained their salient structural characteristics, that is, large live trees arranged in a heterogeneous mixture of groups and individuals (Larson & Churchill, 2012). Meanwhile, simulated fires in the contemporary period produced historically ahistorical forest structures with relatively random and sparse overstories. Though clustered tree regeneration may recover aspects of spatial heterogeneity at some time after fire (Ziegler, Hoffman, Fornwalt, et al., 2017), the overall stocking would likely be well under the natural range of variation for these forests (Safford & Stevens, 2017). This is somewhat counter to findings from studies that reported restorative effects from actual wildfires in long fire‐excluded forests (Jeronimo et al., 2019; Kane et al., 2019; Larson et al., 2013). However, these differences are likely explained by the variation in fire weather in actual wildfires and prefire fuel structures, which are likely more variable than fuel models indicate. While finding of divergent postfire outcomes between historical and contemporary forests is not new (Brown et al., 2008; Taylor et al., 2014), our findings are novel because they explicitly account for differences in the spatial patterns of trees. In doing so, we demonstrated a considerable impact of forests with lower densities and heterogeneous tree arrangements, including sizeable horizontal and vertical fuel gaps, on mitigating fire‐caused tree mortality. Furthermore, our findings serve as quantitative evidence supporting the assertion that historical forests' heterogeneity made them relatively resistant to fire, even under high fire weather conditions (Safford et al., 2012; Show & Kotok, 1924; Stephens et al., 2016).

The lack of canopy gaps in the contemporary period coupled with overall smaller trees allowed for higher intensity fires, which translated to greater predicted tree mortality. These findings can be incorporated in forest restoration strategies that seek to balance seemingly competing objectives, such as high tree canopy cover versus lower forest density (e.g., USFS, 2019). These findings suggest that forest restoration efforts that attempt to mimic historical tree patterns by retaining clumps of high local tree cover, while also creating gaps and isolated individual trees provide a structure that helps reduce wildfire hazard.

### 4.1 Limitations and directions for future research

Virtual experimentation permitted us to simulate potential fire behavior in historical and contemporary forests. This approach overcame a common limitation of using pattern analysis alone to retrospectively infer the effects of processes like fire (Lutz et al., 2018; McIntire & Fajardo, 2009). However, the single set of burning conditions we simulated was narrower than the daily and seasonal variation of fire weather and climate within and across fire seasons. Previous research identified that the interaction between fire behavior and the spatial arrangement of fuels depends on burning conditions (Linn et al., 2013; Parsons et al., 2017). Had we simulated fires under more moderate burning conditions, we might expect fires in 2008 yield more heterogeneous forests, similar to the findings of heterogeneity after fire following actual, moderate fires by Kane et al. (2019). Furthermore, the variability in fire weather over a fire's duration and topographic complexity would be expected to promote heterogeneous residual forest structure. Additional research is needed to understand the mediation of forest structural patterns under a broader set of burning conditions and its implications on the use of prescribed fires and managed wildfires for stand and landscape restoration.

Our study design purposefully excluded impacts from secondary agents of fire‐caused mortality, whose effects on tree spatial patterns can confound the effects from direct fire damage (Yu et al., 2009). However, the approach we used to predict tree mortality (Parsons et al., 2018), while strictly accounting for the effects of direct fire damage, relies on substituting crown consumption for crown scorch in empirical tree mortality equations. This substitution, as well as additional mortality following delayed ecophysiological processes (Hood et al., 2018), may have led to an underprediction of tree mortality, as well as altered patterns of tree mortality (Furniss et al., 2019). Because higher severity fires lead to greater homogeneity of forest structure (Kane et al., 2019; Koontz et al., 2020), any underprediction of tree mortality may have led to overestimated tree spatial heterogeneity after a fire. Additionally, advancements in tree mortality predictive models have incorporated species‐specific response curves relating crown damage to risk of mortality (e.g., Hood and Lutes 2017); these model forms outperform the predictive models from Hood et al. (2007) used here (Grayson et al., 2017), but methodologies to apply these mortality models to output from physical heating models are still in development (Hood et al., 2018). We echo calls for continued research connecting heating and physical damage from fire to tree mortality (Hood et al., 2018; O'Brien et al., 2018). Such efforts will increase the applicability of physics‐based fire models (Parsons et al., 2018).

Finally, it is important to recognize that fires are not the only exogenous agents shaping tree patterns at fine scales. In frequent‐fire forests of the United States, agents such as wind, ice/snow, lightning, animals, bark beetles, and defoliators also shape forest structure (Lundquist & Negron, 2000). Their impacts on tree patterns differ from fire. For example, while creating clustered mortality patterns similar to fire (Addington et al., 2018), mountain pine beetles (*Dendroctonus ponderosae*) preferentially attack moderate to larger individual trees. Since larger trees tend to be aggregated, as in our study, tree mortality patterns resulting from mountain pine beetle may appear as less density‐dependent. Adding further complexity, the impacts of these disturbances are also conditioned on the tree patterns resulting from preceding disturbances (Lundquist & Negron, 2000). Larson and Churchill (2012), for example, suggest that elevated surface fuel accumulation underneath tree groups, which experienced some previously mortality from insects or pathogens would increase the likelihood of fire‐caused mortality. This milieu of biotic and abiotic agents of mortality, in addition to, and interaction with, patterns of fire‐damaged trees, can produce wholesale shifts in patterns of residual living trees, legacy remnants, and tree mortality away from the immediate postfire patterns of live and fire‐killed trees (Furniss et al., 2020). An increased understanding of overlapping disturbances on the formation and modification of tree spatial patterns will aid in the design of restoration treatments and the use of tree spatial patterns to interpret site history.

## 5 CONCLUSIONS

Our study investigated patterns of tree mortality and the consequent patterns of surviving trees following simulated fires in a historical and contemporary mixed conifer forest of the Sierra Nevada. We found that mortality was biased toward smaller diameter trees in the historical period leading to clustered and density‐dependent patterns of tree mortality, while maintaining a diverse range of residual tree groups characteristic of historical dry forests. In the long‐unburned contemporary period, fire‐caused mortality was widespread, resulting in sparse scatterings of trees and small tree groups after fire. Estimated tree mortality and patterns of residual trees were more random and less heterogeneous in the contemporary plots than in either their historical counterparts or the historical range of variability. Our study suggests that high‐severity fires in these, and similar forests, today are unlikely to reestablish the historically characteristic pattern–process linkages. Relying on fire alone to achieve these structural qualities likely requires multiple entries of prescribed fire (Collins et al. 2019) or a fortuitous occurrence of moderate severity wildland fire (Kane et al., 2019). Alternatively, mechanical thinning followed by prescribed fire may achieve these qualities more quickly while leaving less to chance (Knapp et al., 2017). Managed ecological processes and management activities which emulate the characteristics of historical forest structure may enhance resistance to modern wildfires imperiling future forests.

## CONFLICT OF INTEREST

The authors declare no conflict of interest.

## AUTHOR CONTRIBUTION

**Justin Paul Ziegler:** Conceptualization (lead); Formal analysis (lead); Methodology (lead); Resources (equal); Software (equal); Visualization (lead); Writing‐original draft (lead); Writing‐review & editing (lead). **Chad Hoffman:** Conceptualization (equal); Funding acquisition (equal); Investigation (equal); Methodology (equal); Resources (equal); Software (equal); Supervision (equal); Writing‐review & editing (equal). **Brandon Collins:** Conceptualization (supporting); Data curation (equal); Funding acquisition (equal); Project administration (equal); Resources (equal); Supervision (equal); Writing‐review & editing (equal). **Eric Knapp:** Data curation (equal); Writing‐review & editing (equal). **William Mell:** Methodology (equal); Software (equal); Validation (equal); Writing‐review & editing (equal).

## Funding

This work was partially supported by a research partnership between the US Forest Service Pacific Southwest Research Station and UC Berkeley College of Natural Resources (project no. 16‐JV‐11272167‐063), the US Forest Service Pacific Southwest Research Station and Colorado State University (project no. 17‐JV‐11272138‐074), and the US Forest Service Pacific Northwest Research Station and Colorado State University (project no. 19‐JV‐11261987‐085).

## Supporting information

AppendixS1

## Data Availability

Input files, which include the location and sizes of trees, to recreate this study are available from Wildland‐urban interface Fire Dynamics Simulator input files for Pyric Tree Spatial Patterning Interactions in Historical and Contemporary Mixed Conifer Forests, California, USA (https://doi.org/10.5061/dryad.h18931zjp).
