# Supplementary material for: Pyric tree spatial patterning interactions in historical and contemporary mixed conifer forests, California, USA
Source: Ecol Evol. 2020 Dec 25;11(2):820–34. doi: 10.1002/ece3.7084 (PMC7820164; doi:10.1002/ece3.7084)
Supplement: Supplementary file 1 — AppendixS1 [file ECE3-11-820-s001.docx]

**Appendix 1**

In simulating fire in the Wildland-urban interface Fire Dynamics Simulator, we defined the three-dimensional volume being represented, i.e., the domain, including boundary conditions, as well as the coordinates and properties of combustible fuels. The overall domain was composed of 1 m^3^ voxels and measured 850 m *×* 360 m *×* 150 m in the *x*, *y*, and *z* dimensions, respectively. Entering wind at x = 0 m followed a power law function, $u\left( z \right)=u_{20}\times{\frac{z}{20}}^{\frac{1}{7}}$, with speed, *u*, increasing with height above ground level, *z*, according to a defined 20-m wind speed,U_20_, defined in this study as 6 m s^-1^. Boundary conditions on lateral sides, y = 0 m and y = 360 m, and the ceiling, z = 150 m, were mirrored, i.e. free slip and no flux. The outlet boundary condition at z = 850 was open.

Within the domains, we specified the starting fireline and fuel locations. A fireline at *x* = 410 m and *y* = [60*,*300] m freely spread into an area of interest centered about *x* = 610 m and *y* = 180 m. The area of interest contained tree locations and surface fuels based on the respective stem-maps measured in 1929 and 2008 for each of our three plots. Trees were modeled as right circular cones parameterized with the observed height measurements and crown width and crown base height estimated with allometric relationships (Lydersen, unpublished results). Fictional overstory and understory fuels were extended beyond the area of interest to allow the fireline to spread into the area of interest and to develop the wind field upwind of the area of interest. We fit a Thomas point process model using Spatstat v. 1.63-3 (Baddeley and Turner 2005) using the observed tree locations within the area of interest, and then simulated tree locations using this model to randomly assign trees outside of the area of interest. The crown geometry of fictional trees was randomly attributed from the observed trees. We placed timber-litter 3 (Scott and Burgan 2005) surface fuels outside of the area of interest. Material properties of fuels not addressed in the main text are listed in Table A1.

Table A1: Fuel parameters used to populate Wildland urban-interface Fire Dynamics Simulator simulations.

| Fuel type and Parameter | Value |
| --- | --- |
| **Tree crown** |  |
| Surface area/volume (m^-1^) | 4000 |
| Drag coefficient | 0.25 |
| Bulk density (kg m^-3^) | 0.34 |
| Particle density (kg m^-3^) | 520 |
| **Surface fuels- All** |  |
| Drag coefficient | 0.25 |
| Particle density (kg m^-3^) | 510 |
| **Surface fuels- Grass-shrub 2** |  |
| Surface area/volume (m^-1^) | 5992 |
| Load (kg m^-2^) | 0.59 |
| Height (m) | 0.46 |
| **Surface fuels- Shrub 2** |  |
| Surface area/volume (m^-1^) | 5484 |
| Load (kg m^-2^) | 1.90 |
| Height (m) | 0.31 |
| **Surface fuels- Timber-litter 1** |  |
| Surface area/volume (m^-1^) | 5632 |
| Load (kg m^-2^) | 1.50 |
| Height (m) | 0.06 |
| **Surface fuels- Timber-litter 3** |  |
| Surface area/volume (m^-1^) | 5028 |
| Load (kg m^-2^) | 1.21 |
| Height (m) | 0.09 |
